# Novel High Conductive Ceramic Materials Based on Two-Layer Perovskite BaLa_2_In_2_O_7_

**DOI:** 10.3390/ijms232112813

**Published:** 2022-10-24

**Authors:** Nataliia Tarasova, Anzhelika Bedarkova, Irina Animitsa, Ekaterina Abakumova, Ksenia Belova, Hala Kreimesh

**Affiliations:** 1 Institute of High Temperature Electrochemistry of the Ural Branch of the Russian Academy of Sciences, Akademicheskaya St. 20, 620066 Yekaterinburg, Russia; 2 Institute of Hydrogen Energy, Ural Federal University, Mira St. 19, 620000 Yekaterinburg, Russia

**Keywords:** layered perovskite, Ruddlesden-Popper structure

## Abstract

The tasks of quality environmental improvement and the development of new energy sources are very relevant. Hydrogen-operating electrochemical devices are strongly needed innovative ceramic materials with target properties, one of which is a high level of proton conductivity. It this paper, the possibility of proton conductivity in acceptor-doped two-layer compositions based on BaLa_2_In_2_O_7_ was proved for the first time. It was proved that doping leads to an increase in conductivity values up to ~1.5 orders of magnitude. The most conductive is the BaLa_1.9_Sr_0.1_In_2_O_6.95_ composition which demonstrates protonic conductivity value 2 × 10^–5^ S/cm at 450 °C. The acceptor-doped two-layer perovskites is a novel prospective class of proton-conducting materials, and further modification of their composition opens up a new method for the design of electrochemical energy generation devices.

## 1. Introduction

The 21st century poses many challenges for humanity, including the preservation and improvement of the quality of the environment and the creation and development of new energy sources [1,2,3,4]. These two tasks are closely related since the use of fossil fuels, the reserves of which are limited, leads to environmental pollution. Accordingly, the problem of creating an eco-friendly and highly efficient energy source is very relevant. One of the most significant branches of alternative energy is hydrogen energy [5,6,7,8,9,10]. It includes such activity areas as production, storage, transportation and use of hydrogen as an energy carrier. Electrochemical hydrogen-producing and hydrogen-operating devices occupy a significant place in the field of hydrogen energy. Proton-conducting electrolysis cells [11] and solid oxide fuel cells [12,13,14,15,16,17,18,19,20,21,22,23] including proton-conducting fuel cells [24,25,26,27,28,29] can be named as the examples of these devices. Both of them are strongly needed innovative ceramic materials with target properties, one of which is a high level of proton conductivity. In general, electrical conductivity increases with an increase in the concentration of charge carriers and an increase in their mobility. Based on this, the fundamental search for novel materials capable of introducing a large concentration of protons and providing fast protonic transport is a promising direction of modern science.

The traditional high-temperature proton-conducting materials are doped cerates and zirconates of alkali-earth metals [30,31]. The possibility of water uptake into their crystal structure is due to oxygen vacancies, which are given by acceptor doping. The proton concentration in these structures is strictly depends on the concentration of oxygen vacancies and usually does not exceed 0.15–0.20 mol H_2_O per complex oxide formula unit. One of the ways to increase the concentration of oxygen vacancies in the structure is to create multi-sublattice systems in which oxygen vacancies genetically belong to the structure. Examples of such compositions are Ba_4_Ca_2_Nb_2_O_11_ [32] and Ba_2_In_2_O_5_ [33], which contain 0.25 and 0.5 mol of oxygen vacancies per perovskite ABO_3_ formula unit, respectively, and can incorporate the same water concentration. Further increase of water uptake is possible for compositions with another type of crystal structure.

**Figure 1:**
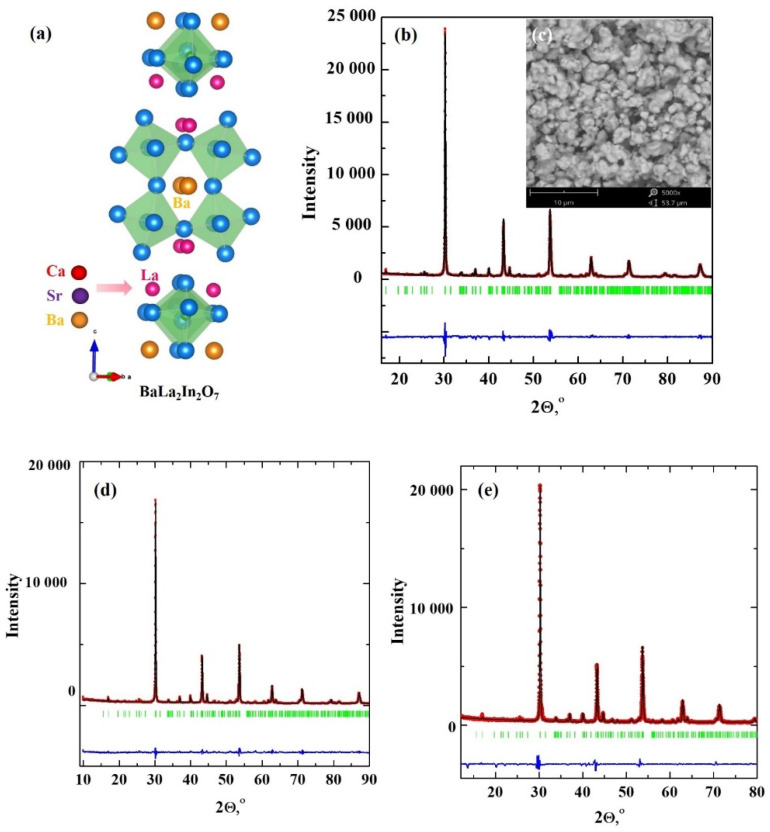
The scheme of acceptor doping of BaLa_2_In_2_O_7_ (**a**), XRD-data (**b**) and SEM-image (**c**) for the composition BaLa_1.9_Sr_0.1_In_2_O_6.95_, XRD-data for the compositions BaLa_1.9_Ba_0.1_In_2_O_6.95_ (**d**) and BaLa_1.9_Ca_0.1_In_2_O_6.95_ (**e**).

Layered perovskites BaLa_n_In_n_O_3n+1_ (n = 1, 2), which can also be named as Ruddlesden-Popper structures, consist of perovskite blocks containing [InO_6_] octahedra and Ba/LaO (n = 1) or LaO (n = 2) layers. The equatorial oxygen atoms bond the octahedra [InO_6_], and axial oxygen atoms are non-bonded with each other. This provides more flexibility to the layered perovskite structure compared with classic perovskite. The doped compositions based on a monolayer (n = 1) BaLaInO_4_ composition can intercalate up to ~2 mol H_2_O per formula unit which is an order of magnitude higher than for acceptor-doped perovskites [34,35]. As previously shown, doped monolayer perovskites BaNdInO_4_ [36,37,38,39,40], BaLaInO_4_ [41,42,43,44,45,46,47,48] and SrLaInO_4_ [49,50,51,52,53] are prospective ionic (oxygen-ionic and protonic) conductors. However, the acceptor doping in the A-sublattices of AA′BO_4_ allows it to achieve higher conductivity values [34,35]. In 2022, two-layer BaLa_2_In_2_O_7_ complex oxide was described as an ionic conductor for the first time [54]. It was shown that the oxygen-ionic and protonic conductivity of BaLa_2_In_2_O_7_ composition is higher than BaLaInO_4_. However, the possibility of proton conductivity in two-layer doped compositions based on BaLa_2_In_2_O_7_ is not yet established. In this paper, the acceptor-doped compositions BaLa_1.9_M_0.1_In_2_O_6.95_ (M = Ca, Sr, Ba) were obtained for the first time (Figure 1a). The possibility of proton transport and the effect of dopant nature on the electrical conductivity were investigated.

## 2. Results and Discussion 

### 2.1. XRD and SEM Characterization

XRD analysis confirms the single phase of all BaLa_1.9_M_0.1_In_2_O_6.95_ (M = Ca, Sr, Ba) obtained compositions, belonging to the space group *P4_2_*/*mnm* (tetragonal symmetry).The results for BaLa_1.9_Sr_0.1_In_2_O_6.95_ are presented in Figure 1b as an example of Rietveld refinement. The increase in the dopant ionic radii ($r_{{\mathrm{La}}^{3+}}$ = 1.216 Å, $r_{{\mathrm{Ca}}^{2+}}$ = 1.18 Å, $r_{{\mathrm{Sr}}^{2+}}$ = 1.31 Å, $r_{{\mathrm{Ba}}^{2+}}$ = 1.47 Å [55]) leads to an increase in the lattice parameters and unit cell volume (Table 1). The data for the undoped BaLa_2_In_2_O_7_ composition (ICSD card number 01-072-6144) are in good agreement with earlier reported data [56,57]. According to SEM investigations, the particles size of obtained compositions is about ~1–3 μm, and agglomerates ~5–10 μm are found also (inset in the Figure 1b). 

### 2.2. Water Uptake 

**Figure 2:**
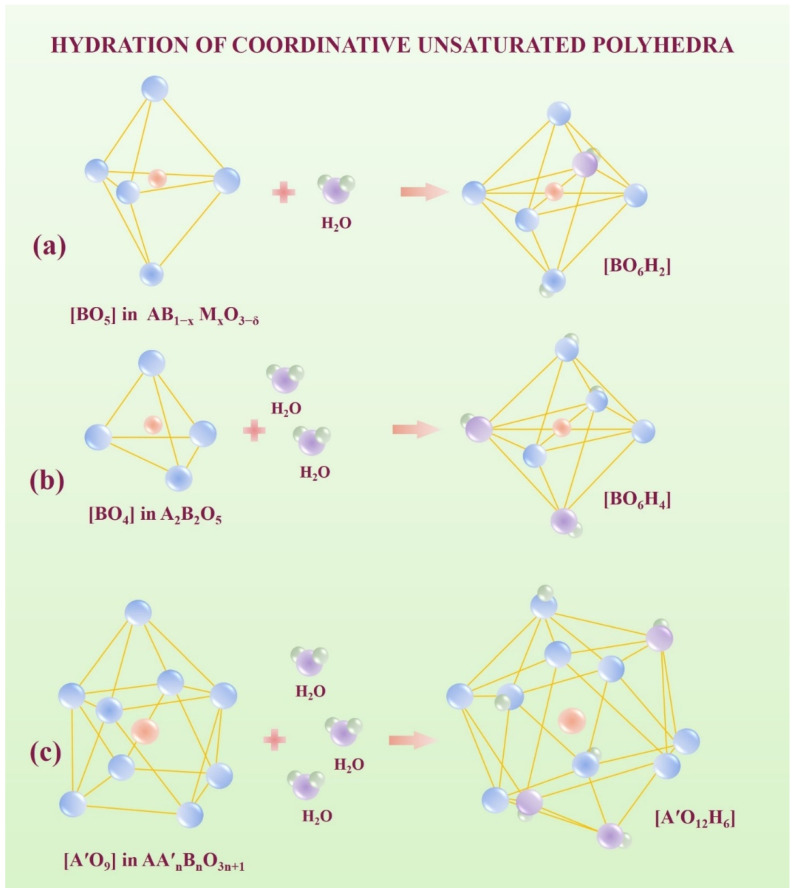
The hydration scheme of coordinative unsaturated polyhedra of acceptor-doped perovskite AB_1−x_M_x_O_3−δ_ (**a**), brownmillerite A_2_B_2_O_5_ (**b**) and layered perovskite AA′_n_B_n_O_3n+1_ (**c**).

The possibility of water uptake by perovskite and perovskite-related structures is determined by various factors. For classical acceptor-doped perovskites, oxygen vacancies are responsible for the appearance of proton defects and when oxygen vacancies are occupied by the oxygen from water molecules, the hydration limit can be reached. For layered RP-perovskites, the possibility of hydration is determined by the size of the rock salt block and the presence of metal ions, which are capable of increasing coordination numbers. It is obvious that the more unoccupied positions the metal has in its coordination environment and the more free space in the structure, the greater the degree of hydration that can be expected. The acceptor-doped classic perovskite AB_1−x_M_x_O_3−δ_ (Figure 2a) or a perovskite-related structure like brownmillerite A_2_B_2_O_5_ (Figure 2b) have polyhedra with a lower coordination number of B-ions. The maximal coordination number of ions located in the B-sublattice is 6, and if it lower then 6, then during hydration the B-cation can increase it from 4 or 5 to 6. Layered perovskites AA′_2_B_2_O_7_ (Figure 2c) have coordinative unsaturated polyhedra [A′O_9_], and ions in the A′-sublattice can theoretically increase the coordination number from 9 to 12. In other words, layered perovskites have ions capable of increasing their coordination number in the A-sublattice. Because A-ions have bigger ionic radii than B-ions, the first have bigger maximal coordination number than the second, and the bigger water uptake during hydration can be predicted for the first. As was shown earlier [34], water uptake for the monolayer compositions based on BaLaInO_4_ was up to 2 mol per formula unit and depends on the size between perovskite layers. So, the theoretically possible 3 mol water uptake per formula unit [A′O_9_] + 3H_2_O → [A′O_12_H_6_] has not been reached due to there not being enough space for the localization of hydroxyl groups. In other words, geometrical factor plays a significant role in the hydration of layered perovskites. 

**Figure 3:**
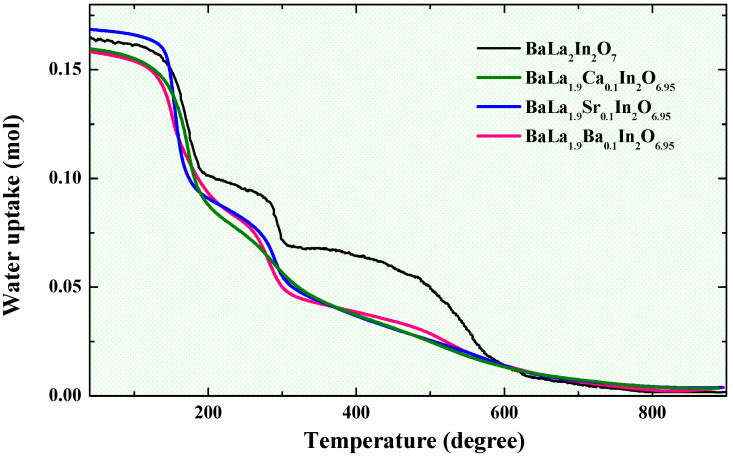
The TG-curves for the investigated compositions.

Monolayer AO(A′BO_3_) and two-layer AO(A′BO_3_)_2_ perovskites are characterized by the ratio between salt layers and perovskite blocks in the structure 1:1 and 1:2, correspondingly. So, the water uptake for the second must be lower than for the first, and the value about 1 mol H_2_O per formula unit can be predicted for the doped two-layer compositions. As previously shown [58], the water uptake for undoped two-layer perovskite BaLa_2_In_2_O_7_ was 0.17 mol, which is ~3.5 times less than for undoped the monolayer BaLaInO_4_ composition (0.62 mol) [41]. Thus, the two-layer perovskite structure has its own specialties concerning of hydration. Figure 3 represents the results of TG measurements for undoped BaLa_2_In_2_O_7_ and doped BaLa_0.9_M_0.1_InO_3.95_ compositions.

As we can see, the shape of TG-curves and water uptake is almost the same for doped and undoped compositions. In the other words, the doping, which is accompanied by the appearance of oxygen vacancies in the structure and the expansion of the inter block space (increase of parameter *c*), does not significantly affect the change in the water uptake. We can assume that increase in coordination number of coordinatively unsaturated atoms (La in BaLa_2_In_2_O_7_) in two-layer structure is difficult compared to structurally flexible monolayer RP-perovskites and water uptake is comparable with water uptake for acceptor-doped classic perovskites (0.15–0.20 mol). Accordingly, the increase in proton conductivity in two-layer perovskites compared to monolayer perovskites can be attributed to an increase in their mobility, and not in their concentration. 

### 2.3. Electrical Conductivity Investigations under Dry (pH_2_O = 3.5 × 10^−5^ atm) Conditions

**Figure 4:**
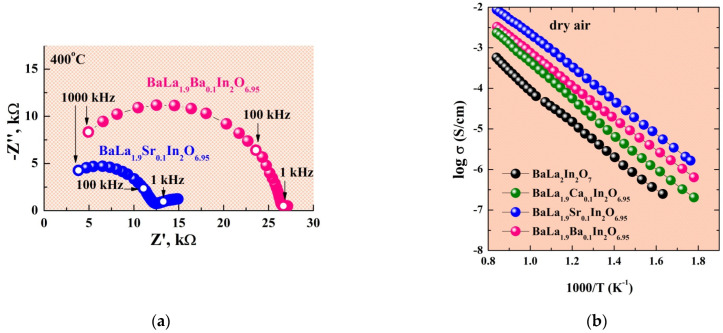
The EIS plots obtained at 400 °C for the compositions BaLa_1.9_Sr_0.1_In_2_O_6.95_ and BaLa_1.9_Ba_0.1_In_2_O_6.95_ (**a**) and temperature dependencies of conductivity for investigated compositions obtained under dry air (**b**).

The impedance spectroscopy method was used for the electrical conductivity measurements. Figure 4a represents the EIS plots for the BaLa_1.9_Sr_0.1_In_2_O_6.95_ and BaLa_1.9_Ba_0.1_In_2_O_6.95_ compositions obtained at 400 °C under dry air. The values of bulk resistance were obtained as the intersection of the semicircle starting from zero coordinates with the Z′ axis. The capacity of these semicircles was about 10^−12^ F. Figure 4b represents the temperature dependencies of electrical conductivity obtained under dry air. As can be seen, doping leads to an increase in the conductivity values for all BaLa_1.9_M_0.1_In_2_O_6.95_ (M = Ca, Sr, Ba) compositions. The most likely reason is the appearance of oxygen vacancies in the structure of doped compositions:

(1) $$2\mathrm{MO}\;\overset{{\mathrm{La}}_{2}O_{3}}{\to }2M_{\mathrm{La}}^{\prime }+2O_{o}^{\times }+V_{o}^{••},$$

where $M_{\mathrm{La}}^{\prime }$—Ca, Sr or Ba-atoms in a La-sites, $V_{o}^{••}$—oxygen vacancy, $O_{o}^{\times }$—oxygen atom in a regular position. The conductivity values increased in the sequence BaLa_1.9_Ca_0.1_In_2_O_6.95_–BaLa_1.9_Ba_0.1_In_2_O_6.95_–BaLa_1.9_Sr_0.1_In_2_O_6.95_, i.e., the Sr-doped composition is most conductive. As previously shown [41], the conductivity values of the monolayer BaLa_0.9_M_0.1_InO_3.95_ (M = Ca, Sr, Ba) compositions increase with the increase of dopant radii and an increase in the space between perovskites blocks. Accordingly, two-layer and monolayer perovskites are characterized by the different regularities. At the same time, it was shown for the classical perovskites La_0.9_M_0.1_InO_3−δ_ (M = Mg, Ca, Sr, Ba) that Sr is a more effective dopant for increasing the oxygen-ionic conductivity [59]. So, we can say that two-layer perovskites BaLa_1.9_M_0.1_In_2_O_6.95_ acquire transport properties that are characteristic of classical perovskites. The increase of n in the formula BaLa_n_In_n_O_3n+1_ leads to a decrease in structural flexibility and the possibility of significant changes in the size of the salt layer during doping. In the other words, two-layer compositions BaLa_1.9_M_0.1_In_2_O_6.95_ are characterized by transport properties of both monolayer AA′BO_4_ and classical ABO_3_ perovskites. 

**Figure 5:**
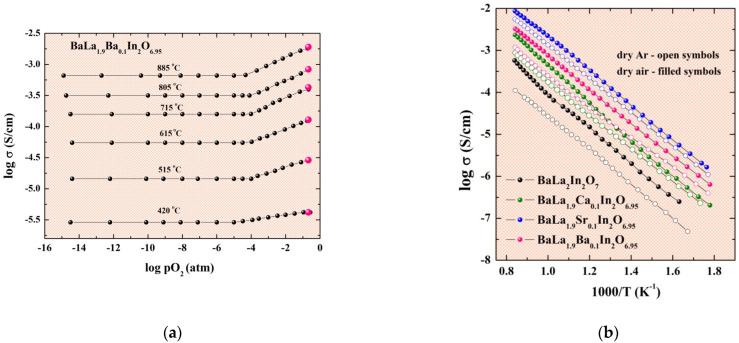
The dependencies of the conductivity values vs. oxygen partial pressure for the composition BaLa_1.9_Ba_0.1_In_2_O_6.95_ and conductivity values from σ–1000/T dependencies under dry air (filled pink symbols) and dry Ar (open pink symbols) conditions (**a**) and temperature dependencies of conductivity for investigated compositions obtained under dry air and dry Ar (**b**).

The conductivity values obtained under variation oxygen partial pressure are presented in the Figure 5a. The nature of conductivity is mixed ionic-hole under dry oxidizing conditions (pO_2_ > 10^–4^ atm):

(2) Vo••+12O2⇔Oo×+2h•,

The area of dominance oxygen-ionic conductivity is observed at pO_2_ < 10^–4^ atm. The conductivity values were obtained under dry Ar conditions also (Figure 5b). As can be seen (open signs in Figure 5a), conductivity values obtained in dry Ar can be considered as the oxygen-ionic conductivity values. Based on this, we can say that oxygen-ionic conductivity is dependent on the dopant ionic radii in the same way as the total conductivity. The higher oxygen conductivity values are observed for the Sr-doped composition. 

### 2.4. Electrical Conductivity Investigations under Wet (pH_2_O = 2 × 10^−2^ atm) Conditions

**Figure 6:**
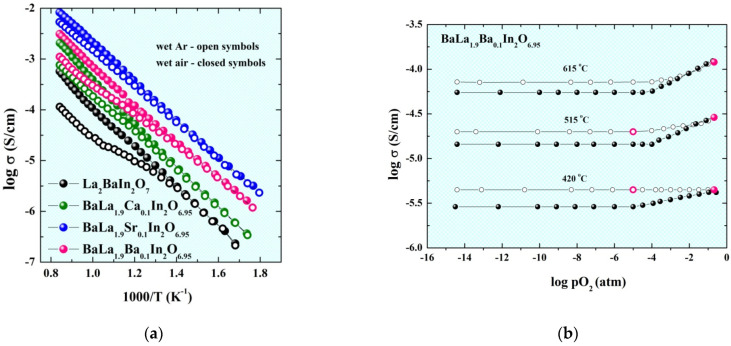
The temperature dependencies of conductivity for the investigated compositions obtained under wet air and wet Ar (**a**) and the dependencies of the conductivity values vs. oxygen partial pressure for the composition BaLa_1.9_Ba_0.1_In_2_O_6.95_ under dry (filled symbols) and wet (open symbols) conditions and conductivity values from σ–1000/T dependencies under wet air (filled pink symbols) and wet Ar (open pink symbols) conditions (**b**).

The temperature dependencies of conductivity obtained under wet conditions are presented in the Figure 6a. 

As we can see, the values at low temperatures (T < 450 °C) obtained under wet air and wet Ar are almost the same for each composition, which is indicates the dominance of proton conductivity in this temperature region. The dependencies of electrical conductivity vs. oxygen partial pressure are presented in Figure 6b. The increase in the proton concentration during lowering temperature leads to the decrease in the hole conductivity:

(3) h•+12H2O+Oo×⇔14O2+(OH)o•

Because of this, the domination of ionic (protonic) transport occurs below ~450 °C which is confirmed by the independence of electrical conductivity on pO_2_. The good comparability between conductivity values obtained under wet Ar (open signs in Figure 6b) and values obtained from σ–pO_2_ dependencies at pO_2_ = 10^–5^ atm should be noted. 

**Figure 7:**
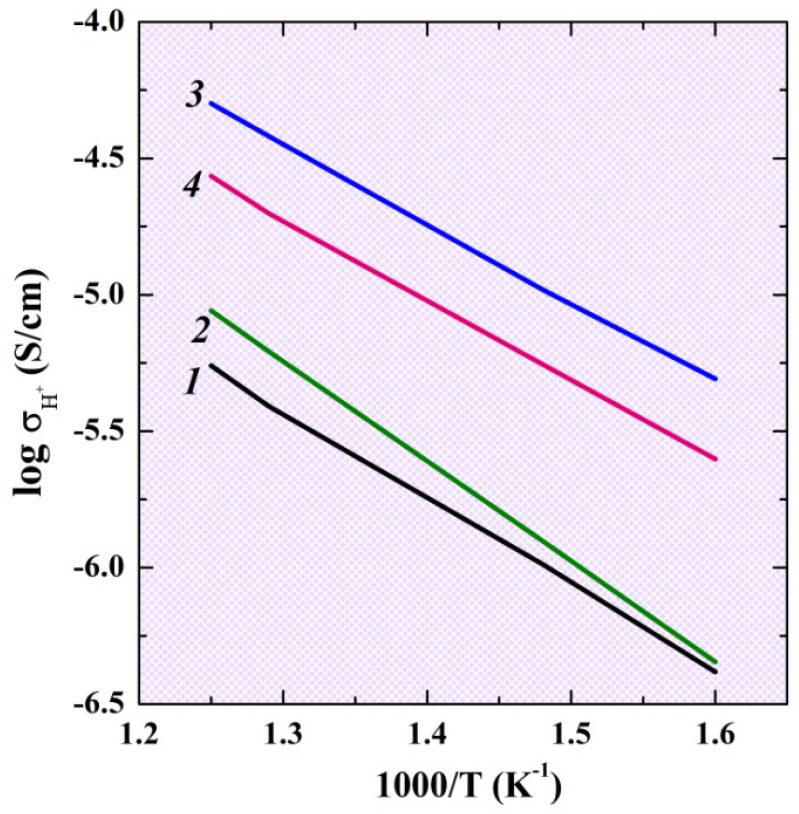
The temperature dependencies of protonic conductivity for the investigated compositions BaLa_2_In_2_O_7_ (1), BaLa_1.9_Ca_0.1_In_2_O_6.95_ (2), BaLa_1.9_Sr_0.1_In_2_O_6.95_ (3), BaLa_1.9_Ba_0.1_In_2_O_6.95_ (4).

Protonic conductivity was calculated as the difference between the conductivity values in wet and dry Ar, and its temperature dependencies for the investigated compositions are presented in the Figure 7. As we can see, doping leads to the increase in the protonic conductivity values, and the most conductive is the Sr-doped composition. Thus, the regularities obtained for the oxygen-ionic conductivity are the same for the protonic conductivity. We can assume that proton transport for two-layer perovskite is provided by jumping over oxygen atoms as it is for the classic perovskites, and the increase in the oxygen mobility leads to an increase in the proton mobility also. 

**Figure 8:**
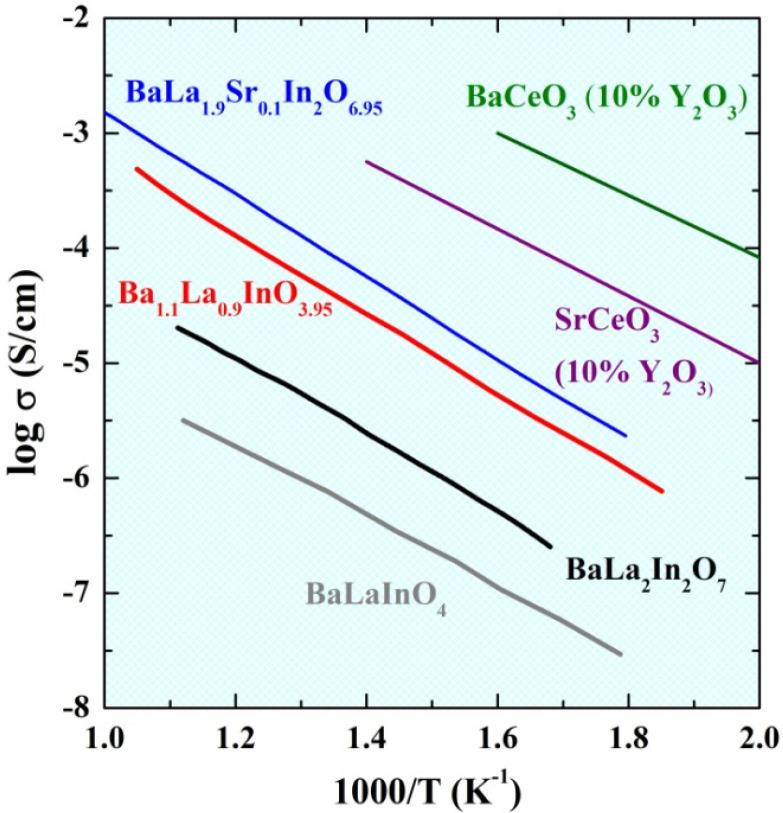
The temperature dependencies of conductivity obtained under wet air for compositions BaLa_1.9_Sr_0.1_In_2_O_6.95_, BaLaInO_4_ [41], Ba_1.1_La_0.9_InO_3.95_ [41], BaLa_2_In_2_O_7_ [54], BaCeO_3_ (10 mol % Y_2_O_3_) [60], SrCeO_3_ (10 mol %Y_2_O_3_) [60].

The comparison of electrical conductivity values obtained under wet air for the undoped BaLaInO_4_ and BaLa_2_In_2_O_7_ and most conductive acceptor-doped monolayer BaLa_0.9_Ba_0.1_InO_3.95_ and two-layer BaLa_1.9_Sr_0.1_In_2_O_6.95_ compositions with such known proton conductors as doped barium and strontium cerates is presented in the Figure 8. As can be seen, two-layer perovskites are characterized by higher conductivity values that monolayer phases, and the acceptor doping can increase the conductivity values up to ~1.5 orders of magnitude under wet conditions. However, the most conductive BaLa_1.9_Sr_0.1_In_2_O_6.95_ composition has lower conductivity values compared with doped barium and strontium cerates. We believe that an increase in the acceptor dopant concentration can increase in the electrical conductivity values, and this direction will be our investigations in the near future.

## 3. Materials and Methods

Two-layer perovskites BaLa_1.9_M_0.1_In_2_O_6.95_ (M = Ca, Sr, Ba) were prepared by the solid state method using carbonates BaCO_3_, SrCO_3_, CaCO_3_ and oxides La_2_O_3_, In_2_O_3_ (99.99% purity, REACHIM, Russia). The calcination was performed from 800 to 1300 °C with 100 °C steps and 24 h temperature treatment on each temperature. 

The X-ray analysis was made using a Bruker Advance D8 Cu K_*α*_ diffractometer (Bruker, Billerica, MA, USA). Morphology of the powder samples was defined by Phenom ProX Desktop scanning electron microscope (SEM) (Thermo Fisher Scientific Inc., Waltham, MA, USA).

The thermogravimetry (TG) and mass-spectrometry (MS) analyses were made using STA 409 PC Netzsch Analyser connected with QMS 403 C Aëolos mass spectrometer (Netzsch, Selb, Germany). The heating of initially hydrated samples was made at the temperature range of 40–1100 °C with a rate of 10 °C/min under a flow of dry Ar. 

The measurements of electrical conductivity were performed by impedance spectroscopy method (Electrochemical Instruments (Elins), Chernogolovka, Russia). The investigations were made from 1000 to 200 °C with 1^o^/min cooling rate under dry air or dry Ar conditions. The dry gas (air or Ar) was produced by circulating the gas through P_2_O_5_ (pH_2_O = 3.5 × 10^−5^ atm). The wet gas (air or Ar) was obtained by bubbling the gas at room temperature first through distilled water and then through saturated solution of KBr (pH_2_O = 2 × 10^−2^ atm). The dependencies of conductivities vs. partial oxygen pressures pO_2_ were obtained by using the electrochemical method for producing different pO_2_ with oxygen pump from Y-stabilized ZrO_2_ ceramic. The values of the resistance were recorded after 3–5 h of equilibrium.

## 4. Conclusions

It this paper, the possibility of proton conductivity in acceptor-doped two-layer compositions based on BaLa_2_In_2_O_7_ was proved for the first time. It was shown that increase in the dopant ionic radii leads to an increase in the lattice parameters and unit cell volume. At dry air conditions, the nature of conductivity is mixed oxygen-hole. Increase in the water partial pressure leads to appearance of protonic contribution in the conductivity under mid and low temperatures. The dominance of proton conductivity is observed below ~450 °C under wet air (pH_2_O = 2 × 10^−2^ atm). Doping leads to an increase in the conductivity values up to ~1.5 orders of magnitude. The most conductive sample is the BaLa_1.9_Sr_0.1_In_2_O_6.95_ composition that exhibits protonic conductivity value 2 × 10^–5^ S/cm at 450 °C. The acceptor-doped two-layer perovskites is a novel prospective class of proton-conducting materials, and further modification of their composition opens up new methods for the design of electrochemical energy generation devices.

## Figures and Tables

**Table 1:** Lattice parameters and unit cell volume for the investigated compositions.

Sample	a, b (Å)	c (Å)	V_cell_ (Å^3^)
BaLa_2_In_2_O_7_	5.914 (9)	20.846 (5)	729.336 (5)
BaLa_1.9_Ca_0.1_In_2_O_6.95_	5.908 (7)	20.861 (7)	728.339 (0)
BaLa_1.9_Sr_0.1_In_2_O_6.95_	5.916 (3)	20.870 (4)	730.518 (3)
BaLa_1.9_Ba_0.1_In_2_O_6.95_	5.921 (0)	20.881 (3)	732.051 (1)

## Data Availability

Not applicable.
